# Long-term preservation of germ cells and gonadal tissues at ambient temperatures

**DOI:** 10.1530/RAF-22-0008

**Published:** 2022-03-21

**Authors:** Pierre Comizzoli, Xiaoming He, Pei-Chih Lee

**Affiliations:** 1 Smithsonian Conservation Biology Institute, National Zoological Park, Washington, District of Columbia, USA; 2 Fischell Department of Bioengineering, University of Maryland, College Park, Maryland, USA

**Keywords:** fertility preservation, biobanking, gametes, gonadal tissues, desiccation, non-cryogenic storage

## Abstract

**Objective:**

To present an overview of different approaches and recent advances for long-term preservation of germ cells and gonadal tissues at ambient temperatures.

**Methods:**

Review of the existing literature.

**Results:**

Preserving viable spermatozoa, eggs, embryos, and gonadal tissues for the long term is critical in human fertility treatment and for the management of animal populations (livestock, biomedical models, and wild species). The need and number of banked germplasms are growing very fast in all disciplines, but current storage options at freezing temperatures are often constraining and not always sustainable. Recent research indicates that structures and functions of gametes or gonadal tissues can be preserved for the long term using different strategies based on dehydration and storage at supra-zero temperatures. However, more studies are needed in rehydration and reanimation of germplasms (including proper molecular and cellular evaluations).

**Conclusions:**

While a lot of research is still warranted to optimize drying and rehydration conditions for each sample type and each species, alternative preservation methods will change the paradigm in fertility preservation and biobanking. It will transform the way we maintain and manage precious biomaterials for the long term.

**Lay summary:**

Living sperm cells, eggs, embryos, and reproductive tissues can be preserved at freezing temperatures for human fertility treatments and used to manage breeding in livestock, laboratory animals, and wild species through assisted reproduction. These cells can be stored in cell banks and demand for them is growing fast. However, current long-term storage options at freezing temperatures are expensive. Instead of using low temperatures, recent research indicates that these cells can be dried and stored above freezing temperatures for an extended amount of time. While a lot of research is still needed to optimize how different samples are dried and rehydrated, alternative methods of preserving cells will make fertility preservation and cell banking easier. It will also transform the way we keep and manage samples for the long term.

## Introduction: values and limitations of cryo-banking in fertility preservation

Preserving viable biomaterials of good quality for the long term is essential in many scientific disciplines. To protect, preserve, or even extend fertility, there is a specific interest in preserving spermatozoa, eggs, embryos, and gonadal tissues (so-called germplasms) in human reproductive medicine, livestock production, laboratory animal management, and wild species conservation (Saragusty *et al.* 2020). The need and demand for reliable and sustainable germplasm storage are exponentially increasing for fertility treatments in humans and animals (in association with the use of assisted reproductive technologies, ART) (Comizzoli *et al.* 2018, Holt & Comizzoli 2021).

Currently, biophysical and biochemical activities can be suspended at freezing temperatures in cells and tissues. This ensures long-term longevity and quality of living biomaterials (Hubel *et al.* 2014, Wolkers & Oldenhof 2021). However, during cryopreservation, germplasms must go through a series of stresses including exposure to toxic cryoprotectant(s), detrimental ice formation during cooling/freezing, possible variations of temperature during storage (risk of accelerated degradation of the samples), and then thawing/warming (risk of devitrification and/or ice recrystallization). Furthermore, sensitivity and response to those stresses vary among species as well as between tissues, cells, organelles, and DNA. We have learned this from years of research in diverse animal models (Comizzoli & Wildt 2013, 2017, Holt & Comizzoli 2021). While vitrification was reported in the mid-1980s to overcome issues related to ice crystal formation in mouse embryos (Rall & Fahy 1985), little attention has been directed to alternative ways for long-term storage of germplasms.

In addition, electrical ultra-cold freezers and liquid nitrogen containers require constant monitoring, complex maintenance, alarm systems, and specialized rooms fitted with backup power and controlled environment. Unfortunately, facilities with sustained supply of electrical power and liquid nitrogen are expensive and not always affordable or readily available in some regions of the world. In addition to possible issues of cross-contaminations in liquid nitrogen (Bajerski *et al.* 2021), cryo-storage systems are prone to failures – from equipment breakdown to human error – which, recently, has led to dramatic sample losses in human fertility clinics and research laboratories (Pomeroy *et al.* 2019, Letterie & Fox 2020). To address the limitations mentioned above, researchers have been exploring for many years alternative solutions to safely store germplasms for later use in fertility preservation programs. The objective of the review is to present different approaches and recent advances toward long-term preservation of germ cells and gonadal tissues at ambient temperatures.

## Principles and different approaches for long-term storage of germplasms at ambient temperatures

### Principles of dehydration

To explore alternative preservation strategies, scientists have been inspired by a vast array of organisms (microbes, fungi, plants, seeds, and animals) that have evolved to survive nearly complete dehydration in nature, sometimes for years or decades. No other strategy in nature is as efficient as dehydration for long-term stabilization. Certain nematodes, tardigrades, insects, and brine shrimp survive extreme cellular water loss via a natural process called ‘anhydrobiosis’ – a term first used by Alfred Giard in 1894 (Keilin 1959, Crowe 2012). Cellular and molecular structures and functions can then be preserved in the dehydrated state above freezing temperatures.

Studies of anhydrobiotic organisms have provided several candidate genes related to the production of ‘xero-protectants’ and conveying tolerance to extreme conditions (Belott *et al.* 2020, Czernik *et al.* 2020, Voronina *et al.* 2020, Anderson & Hand 2021). Unfortunately, desiccation genes or related analogs are not present in vertebrate genomes. It is therefore mandatory to directly supply ‘xero-protectants’ to the cells from vertebrate species before removing the water content (Loi *et al.* 2021).

In addition to slowing down metabolism and producing critical components, one of the keys to reach and survive dry conditions relies on the organisms’ capacity to synthesize and accumulate intracellular disaccharides (mainly trehalose or sucrose) while losing water content (Wolkers & Oldenhof 2021). After introduction to the cells, major advantages of natural disaccharides like trehalose are their low toxicity and high glass-transition temperature (possibility to vitrify at non-freezing temperatures) compared to conventional cryoprotectants such as dimethyl sulfoxide, ethylene glycol, or 1,2-propanediol (Chen *et al.* 2000).

Numerous studies have demonstrated the superior ability of the non-reducing trehalose to stabilize key cellular components, including membranes, proteins, and DNA upon desiccation (Crowe 2012, Zhang *et al.* 2016, Brogna *et al.* 2021, Wolkers & Oldenhof 2021). Three mechanisms have been proposed to explain the desiccation-tolerant properties of trehalose (Hibshman *et al.* 2020). First, the water replacement hypothesis suggests that the disaccharides substitute water molecules during desiccation to form hydrogen bonds with the natural biomacromolecules (such as lipid membrane, proteins, or nucleic acids), which maintains their native 3D/ordered confirmations and structures (Jain & Roy 2009, Golovina *et al.* 2010). As water plays a fundamental role in maintaining protein structure and function, the second hypothesis (water entrapment hypothesis) states that an extremely thin layer of water surrounding surfaces of macromolecules remains entrapped in a shell formed by trehalose to maintain their native 3D/ordered confirmations and structures during desiccation, protecting proteins from damage (Wolkers *et al.* 2010, Olsson *et al.* 2019). Lastly, removal of water leads to increase in trehalose viscosity and transforms it from the liquid state to a glassy state (a process known as vitrification) (Crowe *et al.* 1998). This amorphous glass likely facilitates immobilization of cellular structures and promotes quiescence of enzymatic activities. While the glass-transition property is not unique to trehalose, it has higher glass-transition temperature compared to other disaccharides, potentially allowing stable preservation at higher temperatures, such as ambient temperatures. The three mechanisms are not mutually exclusive and likely operate synergistically to achieve the protective effect of trehalose against desiccation stress. In addition to trehalose, the production of late embryogenesis abundant (LEA) proteins and heat shock proteins (HSPs) have also been observed in a variety of anhydrobiotic organisms. These proteins that form functional 3D conformations upon dehydration, often in conjunction with trehalose, likely act as chaperons to stabilize cellular components (Hincha & Thalhammer 2012, Li *et al.* 2012, Kim *et al.* 2018, Czernik *et al.* 2020).

### Dehydration methods and storage options for germplasms

Note that excellent illustrations of the methods described below are available in recent reviews (Loi *et al.* 2021, Weng 2021).

**Figure 1:**
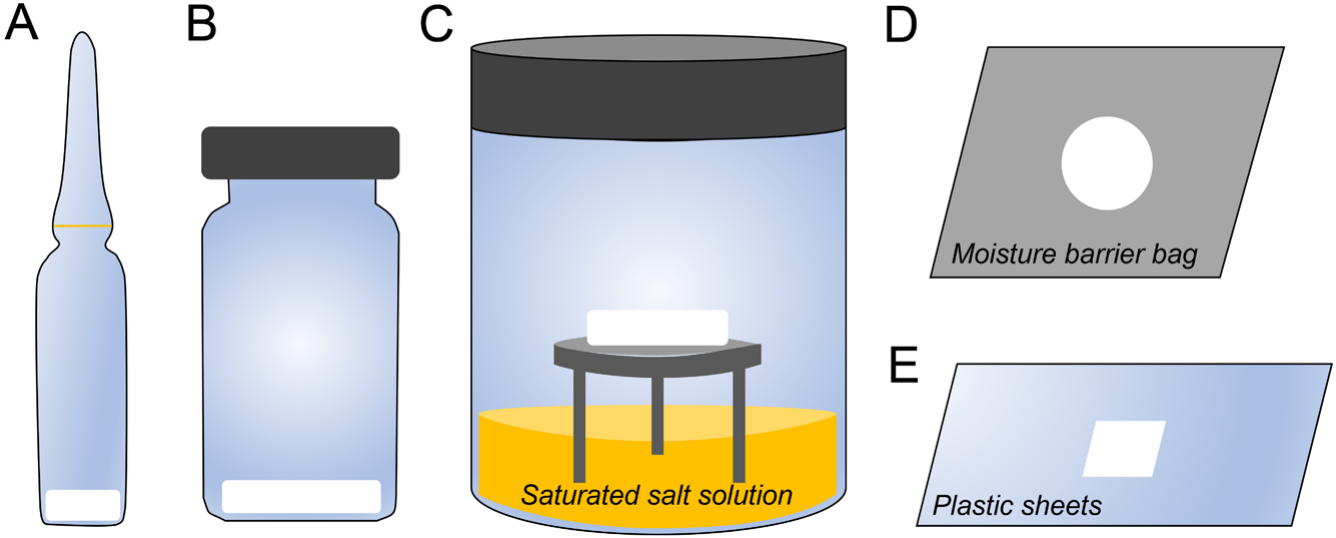
Different types of storage containers for dried samples. Glass ampoule (A), glass vial (B), salt sorption jars (C), moisture-barrier bag (D), plastic sheet (E). All devices are of comparable size, except for the jars that are larger in general.

Lyophilization (freeze-drying) is the most widely used method for germplasm desiccation (Saragusty *et al.* 2020, Loi *et al.* 2021), mainly for sperm cells. The process includes freezing samples followed by primary drying through sublimation of ice at freezing temperature under vacuum and secondary drying through desorption by slowly elevating temperature under vacuum (Weng 2021). Protectants and supplements used in this procedures include trehalose (Martins *et al.* 2007, Sánchez-Partida *et al.* 2008, Ito *et al.* 2019, Shahmoradi *et al.* 2021), buffers like EGTA (Liu *et al.* 2004, Martins *et al.* 2007, Ringleb *et al.* 2013, Palazzese *et al.* 2020) or EDTA (Mercati *et al.* 2020), fetal bovine serum (Choi *et al.* 2011), or media/buffer only (Keskintepe *et al.* 2002, Kwon *et al.* 2004). Sperm cells from many species have been preserved using that method (Patrick *et al.* 2017*a*, Saragusty *et al.* 2020). Embryos or live birth have been obtained from freeze-dried spermatozoa stored at non-freezing temperatures in mouse (Wakayama & Yanagimachi 1998, Kusakabe & Tateno 2011, Ito *et al.* 2019), pig (Kwon *et al.* 2004), monkey (Sánchez-Partida *et al.* 2008), sheep (Olaciregui *et al.* 2017, Anzalone *et al.* 2018, Arav *et al.* 2018), horse (Choi *et al.* 2011), or cattle (Keskintepe *et al.* 2002). All studies had to use intra-cytoplasmic sperm injection (ICSI) as sperm cells are not motile after rehydration. Most common storage containers for lyophilized sperm cells are glass ampoules/vials (Fig. 1). Recently, it has been demonstrated in mouse that freeze-drying on weighing paper and then storing in between plastic sheet had comparable outcomes, which further simplifies storage (Ito *et al.* 2021). Freeze-drying of sperm cells in trehalose has also been successful in humans (Gianaroli *et al.* 2012, Keskintepe & Eroglu 2021). Recently, partial freeze/dried human spermatozoa was successfully rehydrated and utilized for ICSI, leading to the production of normal euploid human blastocysts (Alexandrova *et al.* 2020).

Regarding the female gamete, freeze-dried porcine nuclei (germinal vesicles) can resume meiosis after transfer into an enucleated oocyte (Dang-Nguyen *et al.* 2018). Further analysis demonstrated that a small portion of dried germinal vesicles stored in glass vial at non-freezing temperatures retained intact nuclear envelope and/or DNA. Freeze-drying of ovarian tissue has recently been attempted in sheep. Results showed high levels of RNA degradation and morphological alteration (Bebbere *et al.* 2021). Nonetheless, it provided clues for subsequent improvement and storage options. Currently, there are no reports on freeze-drying of embryos or testicular tissues yet.

Passive air drying or evaporative drying have been attempted in sperm cells and germinal vesicles. Long-term storage after evaporative drying was successful in mouse sperm cells (Li *et al.* 2007). Air drying and ambient temperatures also induce conformational changes of nucleic acids and stallion sperm chromatin in trehalose preservation formulations (Brogna *et al.* 2021). Comparable observations about the loss of DNA integrity have also been reported in air-dried llama spermatozoa (Carretero *et al.* 2020). On the female side, cat germinal vesicles that were air-dried in trehalose and stored at 4°C for up to 8 weeks largely retained DNA and membrane integrity. A portion of these nuclei were capable to resume meiosis after transfer to fresh cytoplasts (Graves-Herring *et al.* 2013).

Convective drying is a different method that utilizes dry gas (nitrogen) to facilitate active water evaporation and vapor removal. It is one of the earliest methods tested to bypass freezing to achieve sample desiccation. The approach has been used for desiccation of mouse spermatozoa, which resulted in fetus production (Bhowmick 2003, McGinnis 2005, Liu *et al.* 2012). The spermatozoa in these studies were dried on glass slides and stored either in vacuum-sealed bags (Bhowmick 2003, McGinnis 2005) or salt-sorption jars (Liu *et al.* 2012) at 4°C or ambient temperatures (Fig. 1).

Microwave-assisted drying is one of the most recent strategies to accelerate water evaporation while staying within physiological temperatures. It was first explored to desiccate live mammalian cells (mouse macrophage cells) in 2008, showing viability after rehydration (Chakraborty *et al.* 2008). Drying kinetics and sample uniformity were thoroughly characterized (Chakraborty *et al.* 2008, Cellemme *et al.* 2013). It was later translated to the cat spermatozoa, with morphology and DNA integrity being maintained after drying on coverslips. Even after immediate rehydration, developmental potential was reduced (Patrick *et al.* 2017*b*). In recent studies using storage in moisture-barrier bags (Fig. 1) at −20°C, DNA integrity was unchanged in the first 3 months and only moderately decreased after longer storage (5–16 months). Developmental potential was sustained after up to 16 months of storage (Lee *et al.* 2021). Other studies focused on the cat germinal vesicle that could be dried on glass fiber filter paper to a moisture level that was compatible with supra-zero temperature storage. DNA integrity was mostly maintained after storage for up to 8 weeks at either 4°C or ambient temperatures in moisture-barrier bags (Elliott *et al.* 2015). Epigenetic alteration (decreased H3K4me3) was observed after germinal vesicle drying as well as an increase in structural damage of nuclear envelope and chromatin but to a lesser extent compared to cryopreservation (Lee & Comizzoli 2019). The feasibility of applying this drying technique to gonadal tissue has also been explored. Drying of cat ovarian tissue showed limited impact on morphology but altered transcriptional activity and gene expression (Lee & Comizzoli 2019, Lee *et al.* 2019, Amelkina & Comizzoli 2020). Recently, drying of cat testicular tissues showed that structural integrity and cell viability could be maintained at an acceptable level (Silva *et al.* 2020).

Other dehydration methods have been explored but there are no reports on germplasms or even live cells yet. Spin drying combines convective evaporation with water removal by centrifugal force to form an ultra-thin layer of samples for achieving rapid drying. During passive drying in trehalose droplets, a thin glassy film may form at the trehalose/air interface to dramatically slow down the evaporative desiccation of trehalose solution (He *et al.* 2008, Chakraborty *et al.* 2011). The method was first developed to overcome this caveat and provide more homogenous desiccation and effective drying was then confirmed by Raman spectroscopy (Abazari *et al.* 2014). Spin drying of mammalian cells (CHO cells) retained membrane integrity in >95% of cells (Chakraborty *et al.* 2011); however, functional survival of live cells has not been reported. Lastly, light-assisted drying (LAD) utilizes near-infrared laser light to facilitate samples drying. Initial reports suggested maintenance of protein functionality after drying (Young *et al.* 2018). The authors also showed that LAD-processed samples largely remained in glassy state after storage at ambient temperatures at low relative humidity (Furr *et al.* 2020).

Overall, these encouraging results clearly show that structures and, more importantly, functions of gametes and gonadal tissues can be suspended in trehalose glass after desiccation and potentially be preserved for the long term at supra-zero temperatures. In the meantime, we also learned that there is still a need for environmental control during storage (temperature and relative humidity levels). As mentioned above, storage containers for ambient temperature storage vary according to the methods (Fig. 1). Glass ampoules (Kamada *et al.* 2018) or glass vials (Dang-Nguyen *et al.* 2018) have been traditionally used to store lyophilized germplasms. The main inconvenience is that ampoules are breakable. Salt-sorption jars have been experimented to control the percentage of relative humidity (Liu *et al.* 2012), but their size is not practical for long-term storage. More recently, moisture-barrier bag (Elliott *et al.* 2015, Lee *et al.* 2021) or plastic sheets (Ito *et al.* 2021) have been used. Besides the advantage of gaining space for storage, they also are easier to ship from one location to the other.

## Research directions to optimize germplasm preservation at ambient temperatures

### Studies on rehydration and recovery from the desiccation stress

So far, most studies focused on optimizing the drying process to reduce the damage, and little is known about rehydration conditions, which is a critical step (Loi *et al.* 2021). Commonly, rehydration is achieved by simply adding the volume of water lost back to the sample. One study reported that stepwise rehydration with serial dilution of trehalose after microwave-assisted drying of cat germinal vesicle did not seem to mitigate drying-induced epigenetic alteration (Lee & Comizzoli 2019). Recent studies in anhydrobiosis provided valuable insight into the molecular regulation of the recovery process. For instance, a high-throughput mass spectrometry analysis in Chironomus discovered that, while trehalose is crucial during desiccation, glucosamine appears to be essential for recovery (Thorat *et al.* 2017). Transcriptome and/or proteomic analysis revealed that several DNA repair systems including homologous recombination, nucleotide excision repair, non-homologous end joining are active during rehydration/recovery phase in an anhydrobiotic cell line and a desiccation-resistant bacterium (Ujaoney *et al.* 2017, Yamada *et al.* 2018). Certain HSPs also are upregulated by rehydration processes in fly pupae and springtails, suggesting distinct roles (Hayward *et al.* 2004, Sørensen *et al.* 2010).

### Other research needs

**Figure 2:**
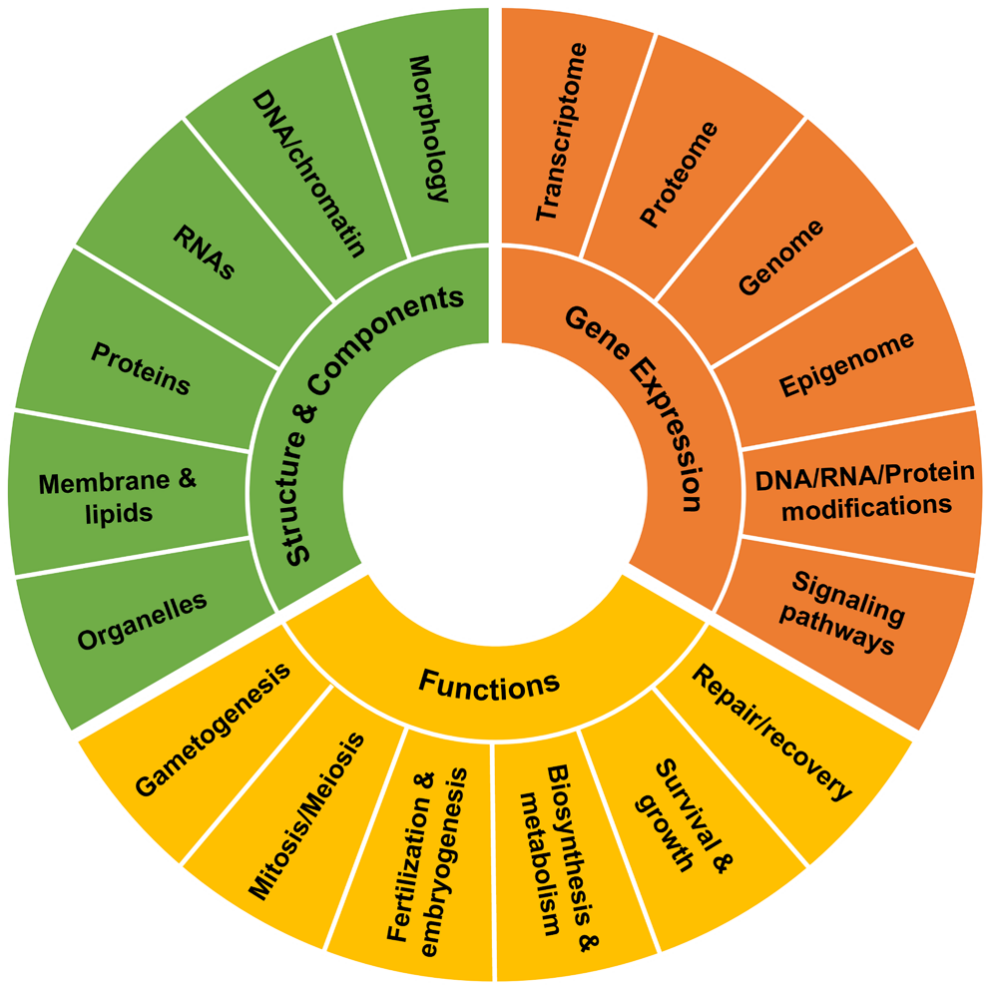
Full list of required evaluations for rehydrated samples.

Despite encouraging advances in storage at ambient temperatures, more research is needed. As mentioned above, the scientific and technical evidence of an optimal dehydration method is missing (including the species-specificities of the approaches). Although natural ‘anhydrobiosis’ is inspiring, none of the small organisms mentioned earlier undergo freezing followed by low-pressure sublimation of ice. Thus, there is an urgent need to optimize the dehydration process and storage containers with devices adapted to the type and size of each sample for each animal species. A comprehensive list of necessary evaluations in rehydrated samples is provided in Fig. 2. So far, most studies have focused on structures/components and functions of germplasms although biosynthesis and metabolism have not been thoroughly explored. Too little research has been conducted on gene expression (Fig. 2), which should be one of the highest priorities in the coming years to develop optimal protocols for different species.

Collaborative efforts with bioengineers have been fruitful to develop dehydration and storage methods. Continued work at the intersection of these disciplines will lead to the best solutions for germplasms. Although motile sperm cells would be desirable (to allow* in vitro* fertilization), progresses in ICSI are made rapidly as shown by the increasing success of embryonic development following injection of dried/rehydrated spermatozoa. Even minor technical improvements affect the efficiency dramatically (Palazzese *et al.* 2020). Further research in experimental animal models also is warranted before translating new knowledge to other species, including humans. While there might be a growing interest in human reproductive medicine in adopting dry storage for human spermatozoa (Gianaroli *et al.* 2012), many more data are required on the production of live offspring with freeze-dried spermatozoa, including their health later in life, even at the epigenetic/genetic level to exclude long-term side effects on DNA caused by desiccation and storage at ambient temperatures (Fig. 2). Lastly, studies on germplasms in the following areas will help to make progress faster: trehalose delivery, including through nanoparticles (Rao *et al.* 2015, Zhang *et al.* 2019); adaptation of drying technologies to large and complex biological samples (tissues, organs); and genome-wide evaluations (transcriptome, epigenome).

## Conclusions and future perspectives about operations of biobanks at ambient temperatures for humans or animal species

Regardless of the drying and storage approaches that are chosen for fertility preservation, we will still have to ask the same essential questions to any new emerging banking effort: What are we storing? Why are we storing it? What storage container are we using? How many do we want to store? For how long? However, storage at ambient temperatures will lead to different biobanking logistics and operations in terms of processes, maintenance, and curation (Comizzoli *et al.* 2022). Desiccating and storing germplasms at ambient temperatures would be highly advantageous. It would decrease the costs related to processing and storage of samples by simplifying the preservation methods, reducing the need for specialized space/infrastructures, and avoiding liquid nitrogen purchase. Biosecurity (prevention of pathogen transmission) of storage at supra-zero temperatures in individual containers should be higher than for samples placed within the same liquid nitrogen vat. Transport of biomaterials between locations will be easier, with patients even having the option of at-home-storage for their own samples. Lastly, while moisture content will have to be maintained to a low level to prevent degradation during storage, samples will be more resilient to variations of temperatures than frozen samples.

Even though there will be less constraints in terms of the location, new storage facilities at ambient temperatures will still require environmental control, sample accessioning, and safety. New sample holders and identification/ labeling methodologies will also be needed for dried samples. In sum, a whole set of standard operating procedures will have to be developed. As mentioned, ambient temperature storage will also help to develop the concept of de-centralized biobanks (or home-storage) that involves less liability than centralized biobanks. Samples would be closer to the end-users and could be easily stored for a short duration. However, new sets of ethical aspects and issues of proper use (risk of parallel markets) may have to be anticipated.

## Declaration of interest

The authors declare that there is no conflict of interest that could be perceived as prejudicing the impartiality of this review.

## Funding

For P C and P C L, this publication was supported by the Office of The Director, National Institutes of Health http://dx.doi.org/10.13039/100000002 under Award Number R01OD023139. The content is solely the responsibility of the authors and does not necessarily represent the official views of the National Institutes of Health http://dx.doi.org/10.13039/100000002. X H is supported by NSF CBET-1831019 and NIH R01EB023632.

## Author contribution statement

P C conceived the idea for the article, undertook the literature search, wrote the manuscript, and approved the manuscript for submission. X H conceived the idea for the manuscript, undertook editorial changes, and approved it for submission. P C L conceived the idea for the article, undertook the literature search, wrote sections of the manuscript, developed the figures, and approved the manuscript for submission.
